# MicroRNA‐411‐3p inhibits bleomycin‐induced skin fibrosis by regulating transforming growth factor‐β/Smad ubiquitin regulatory factor‐2 signalling

**DOI:** 10.1111/jcmm.17055

**Published:** 2021-11-15

**Authors:** Ziyan Zhang, Xuemin Gao, Yang He, Yumeng Kang, Fuyu Jin, Yaqian Li, Tian Li, Zhongqiu Wei, Shifeng Li, Wenchen Cai, Na Mao, Shan Wang, Heliang Liu, Fang Yang, Hong Xu, Jie Yang

**Affiliations:** ^1^ Department of Dermatology Affiliated Hospital of North China University of Science and Technology Tangshan Hebei Province People’s Republic of China; ^2^ School of Public Health Hebei Key Laboratory for Organ Fibrosis Research North China University of Science and Technology Tangshan Hebei Province People’s Republic of China

**Keywords:** miR‐411‐3p, skin fibrosis, Smurf2, transforming growth factor‐β1

## Abstract

Skin fibrosis, which is characterized by fibroblast proliferation and increased extracellular matrix, has no effective treatment. An increasing number of studies have shown that microRNAs (miRNAs/miRs) participate in the mechanism of skin fibrosis, such as in limited cutaneous systemic sclerosis and pathological scarring. The objective of the present study was to determine the role of miR‐411‐3p in bleomycin (BLM)‐induced skin fibrosis and skin fibroblast transformation. Using Western blot analysis and real‐time quantitative polymerase chain reaction assess the expression levels of miR‐411‐3p, collagen (COLI) and transforming growth factor (TGF)‐β/Smad ubiquitin regulatory factor (Smurf)‐2/Smad signalling factors both in vitro and in vivo with or without BLM. To explore the regulatory relationship between miR‐411‐3p and Smurf2, we used the luciferase reporter assay. Furthermore, miR‐411‐3p overexpression was identified in vitro and in vivo via transfection with Lipofectamine 2000 reagent and injection. Finally, we tested the dermal layer of the skin using haematoxylin and eosin and Van Gieson's staining. We found that miR‐411‐3p expression was decreased in bleomycin (BLM)‐induced skin fibrosis and fibroblasts. However, BLM accelerated transforming growth factor (TGF)‐β signalling and collagen production. Overexpression of miR‐411‐3p inhibited the expression of collagen, F‐actin and the TGF‐β/Smad signalling pathway factors in BLM‐induced skin fibrosis and fibroblasts. In addition, miR‐411‐3p inhibited the target Smad ubiquitin regulatory factor (Smurf)‐2. Furthermore, Smurf2 was silenced, which attenuated the expression of collagen via suppression of the TGF‐β/Smad signalling pathway. We demonstrated that miR‐411‐3p exerts antifibrotic effects by inhibiting the TGF‐β/Smad signalling pathway via targeting of Smurf2 in skin fibrosis.

## 1 INTRODUCTION

Skin fibrosis is a clinical manifestation of skin diseases. The main pathological characteristics are increased extracellular matrix (ECM) and fibroblast proliferation. However, the aetiology of fibrotic skin diseases is not well understood, and there are no effective curative therapies. Skin fibrosis poses a substantial problem to the socio‐economic and medical health systems. In recent years, an increasing number of studies have revealed that non‐coding RNAs (ncRNAs) play an important role in various diseases, including cancers and skin fibrosis.^1^, ^2^, ^3^, ^4^ Small ncRNAs called microRNAs (miRNAs/miRs) are involved in skin fibrosis.^5^, ^6^ MiRNAs, which have 18–24 nucleotides, repress target mRNAs by binding to the 3′ untranslated region (3′‐UTR). One miRNA can regulate various mRNAs and may be involved in multiple signalling pathways. In some cancers, including renal cell carcinoma, cervical cancer and colorectal cancer, miR‐411 is downregulated. Overexpression of miR‐411 regulates related target genes, thereby attenuating proliferation and migration while promoting apoptosis of cervical cancer cells.^7^, ^8^, ^9^ In deep venous thrombosis, injecting miRNA‐411 mimic can inhibit vein wall fibrosis by regulating matrix metalloproteinase‐2 with hypoxia‐inducible factor‐1α.^10^ Our previous research found that miR‐411‐3p was downregulated in rat silicosis, and overexpression of miR‐411‐3p suppressed collagen hyperplasia and fibroblast migration and proliferation.^11^

Bleomycin (BLM) can induce transforming growth factor (TGF)‐β1 expression in lung fibroblasts in concentration‐ and time‐dependent manners.^12^ As previously determined, TGF‐β is a profibrotic factor.^13^ Smad ubiquitin regulatory factor (Smurf)‐2, an E3 ubiquitin ligase, has been shown to negatively affect TGF‐β signalling.^14^ Smurf2 has a high binding capacity with Smad2, which may be one of the preferred targets in TGF‐β/Smad signalling. The complex of Smad4 with either Smad7 or Smad2 can be degraded by binding to Smurf2.^15^ Moreover, in the nucleus, Smurf2, which is complexed with Smad7, leads to TGF‐β receptor (TGF‐β RI) activation. In recent years, Sim et al. found that two phosphorylation sites on Smurf2, Y314 and Y434, exert inhibitory effects on Smurf2 and Smad7.^16^ Tetratricopeptide repeat domain‐3 expression can promote TGF‐β1 induction of the epithelial‐mesenchymal transition (EMT) and myofibroblast differentiation by reducing Smurf2 ubiquitylation/proteasomal degradation, following which the inhibition of Smurf2 by Smad2/3 is released.^17^ In cardiac fibrosis, trimethylamine N‐oxide can exacerbate the expression of smooth muscle actin‐α (α‐SMA) and collagen I (COLI) by inhibiting the ubiquitination of TGF‐β RI and Smurf2, thereby enhancing the TGF‐β/Smad2 signalling pathway.^18^

Different studies have shown that Smurf2, as a target, can be regulated by diverse miRNAs in various diseases,^19^, ^20^ and several target prediction databases have also demonstrated similar findings. MiR‐497 and miR‐195 combine with the 3′‐UTR of Smurf2, and upregulation of miR‐497 and miR‐195 reduces lung cancer cell colonization ability and invasion by suppressing the ubiquitination of TGF‐β RI via inhibition of the target gene Smurf2.^19^ In chronic asthmatic mice, overexpression of miR‐485 inhibits the proliferation and promotes the apoptosis of air smooth muscle cells (ASMCs) by reducing Smurf2‐controlled TGF‐β/Smad3 signalling.^20^ Our previous study found that an miR‐411‐3p mimic can attenuate silicosis in mice and inhibit TGF‐β1‐induced transformation of lung fibroblasts to myofibroblasts by binding to Smurf2.^11^

The objective of the present study was to determine the role of miR‐411‐3p in BLM‐induced skin fibrosis and skin fibroblast transformation. The mechanism between miR‐411‐3p and the TGF‐β/Smad signalling pathway was found to be influenced by miR‐411‐3p and Smurf2.

## 2 MATERIALS AND METHODS

### 2.1 Animal experiments

All male C57BL/6 mice (approximately 20 g and 6 weeks old) were provided by Vital River Laboratory Animal Technology Co., Ltd. The animal experiments were approved by the Ethics Committee for Animal Experimentation of North China University of Science and Technology (2013–038) and conformed to the guidelines set by the National Institutes of Health.

The mice were divided into three groups: (1) agomir‐negative control (NC), (2) BLM + agomir‐NC, and (3) BLM + agomir miR‐411‐3p (*n* = 6 mice per group). BLM was dissolved in saline solution at a concentration of 1 mg/ml, and 100 μl was intradermally injected into the shaved dorsal skin of each mouse every day for 4 weeks.^21^ Agomir‐NC or agomir miR‐411‐3p was dissolved in 100 μl saline solution,^22^ and 1 nmol (20 μl) was intradermally injected into the shaved skin of each mouse beginning with the first dosing of BLM. The injection of agomir‐NC or agomir miR‐411‐3p was performed once a week four times after the first injection of BLM. The mice were administered a final injection of BLM and fed for another 24 h. Then, the mice were killed by anaesthetic injection. The shaved dorsal skin was harvested and fixed in 4% paraformaldehyde for histological analysis.

### 2.2 Cell experiments

Primary fibroblasts were acquired from the back skin of newborn mice and cultured in low‐glucose Dulbecco's Modified Eagle's Medium (BISH1734; Biological Industries, Beit HaEmek, Israel) with 10% foetal bovine serum (50216; Bovegen Biologicals, Melbourne Australia) and 1% penicillin‐streptomycin in 25 cm^2^ culture flasks, which were placed in an incubator with 5% CO_2_ at 37°C. The concentration of BLM (19051511; Nippon Kayaku) was 3 mU/mL, which was incubated together with FBS.^12^ MiRNAs and small interfering RNAs (siRNAs; Ribobio) were transfected with Lipofectamine 2000 reagent (11668‐019; Invitrogen, Thermo Fisher Scientific) at a final concentration of 50 nM according to the transfection protocol. The sequences of the miRNAs and siRNAs are shown in Table S1.

### 2.3 Western blot analysis

Secondary fibroblasts were treated with BLM, miRNA or siRNA, following protein isolation using radioimmunoprecipitation assay lysis buffer. The concentration of total protein was measured using bicinchoninic acid reagent (70‐PQ0012; MultiSciences Biotech Co., Ltd., Hangzhou, China). The primary antibodies used were COLI (BA0325; 1:1000; BOSTER), TGF‐β1 (ARG56894, 1:1000; Arigobio) and TGF‐RI (A16983, 1:1000; ABclonal). Membranes were washed three times using tris‐buffered saline‐Tween 20 for 10 min each and then incubated with the secondary antibody (H + L, 074‐1506/074‐1806; Kirkegaard and Perry Laboratories). Tubulin‐α (GTX112141, 1:8000; Genetex) was used as an internal reference.

### 2.4 Real‐time quantitative polymerase chain reaction (RT‐qPCR)

Using TRIzol reagent (Ambion), total RNA was extracted from secondary fibroblasts and mouse skin. Reverse transcription (RK20402; ABclonal) was performed according to the manufacturer's protocol. For RT‐qPCR, 2 µl of complementary DNA with 2× SYBR qPCR Mix (RK20404; ABclonal) was used. The primer sequences were as follows: (1) mouse *Col1a1*, forward: GCTCCTCTTAGGGGCCACT, reverse: CCACGTCTCACCATTGGGG; (2) mouse *Gapdh*, forward: CCTGCACCACCAACTGCTTA, reverse: GCCCCACGGCCATCACGCCA; (3) mouse *Smurf2*, forward:GTGAAGAGCTCGGTCCTTTG, reverse: AGAGCCGGGGATCTGTAAAT; (4) mouse *Tgf*‐*β1*, forward: CAATTCCTGGCGATACCTCAG, reverse: GCACAACTCCGGTGACATCAA. *GAPDH* was used as a reference.

MiRNAs were reverse transcribed using other transcription kits (K1622; Thermo Fisher Scientific or ZR102; ZOMANBIO). The miRNA primers used were as follows: (1) Bulge‐loop™ U6 RT primer (no. ssD0904071008); (2) Bulge‐loop™ miR‐411–3p RT primer (no. miR8002824); (3) Bulge‐loop™ miR‐411–3p forward primer (no. miR8002825); (4) Bulge‐loop™ U6 forward primer (no. ssD0904071006); (5) Bulge‐loop™ miR‐411–3p reverse primer (no. ssD089261711); and (6) Bulge‐loop™ U6 reverse primer (no. ssD0904071107). U6 was used as the reference for miR‐411‐3p. Gene expression was calculated using the 2^−ΔΔCT^ method.

### 2.5 Luciferase reporter assay

Smurf2‐3′‐UTR wild‐type (Wt) plasmid or Smurf2‐3′‐UTR‐mutant (Mut) plasmid was synthesized and cloned into the pmirGLO reporter vector (Ribobio) containing the firefly luciferase gene and the control Renilla luciferase gene. Human embryonic kidney (HEK)293T cells were co‐transfected with the luciferase reporter vector and miR‐411‐3p negative control or miR‐411‐3p mimic using Lipofectamine 2000 reagent (11668‐019; Invitrogen, Thermo Fisher Scientific). At 48 h after transfection, luciferase activity was measured using the Dual‐Luciferase Reporter Assay System (E1910; Promega).

### 2.6 In situ hybridization of miR‐411‐3p

Paraffin‐embedded tissue was dewaxed and rehydrated routinely. Tissue sections and fibroblasts were exposed to nucleic acid fragments containing pepsin at 37°C. Next, the tissue sections and fibroblasts were incubated with hybridization solution (MK10606; Boster Biological Technology, Wuhan, China) containing the miR‐411‐3p probe (5′‐GGTTA GTGGACCG TGTTACATA‐3′) at 38–42°C overnight. The sections were blocked at 37°C for 30 min and incubated with a digoxin‐labelled biotinylated antibody (MK10606; Boster Biological Technology) for 2 h at 37°C. After washing several times, the sections were incubated with cyanine 3‐labelled streptavidin‐biotin complex for 1 h at 37°C. Finally, the fluorescence intensity was observed using a microscope.

### 2.7 Histology and immunofluorescence

Skin tissues were stained with haematoxylin and eosin (Sigma‐Aldrich) and Van Gieson's stain (BA4084; Baso Diagnostics Inc.) in order to observe the morphology of the skin and measure the expression of collagen, respectively. Fibroblasts were incubated with the primary antibody of COLI (BA0325; 1:200; Boster Biological Technology) at 4°C overnight. After incubation, the specimens were washed three times and then incubated with a fluorescent‐labelled secondary antibody. Tissues and cells were visualized using a microscope.

### 2.8 Phalloidin staining

The fibroblasts were treated with BLM (3 mU/mL) for 12 h, fixed with 4% paraformaldehyde for 1 h, and washed three times with phosphate‐buffered saline. Before phalloidin staining, the fibroblasts were incubated with 0.1% Triton X‐100 (9002‐93‐1; Sigma‐Aldrich) for 5 min. A final concentration of 50 μg/mL fluorescein isothiocyanate‐labelled phalloidin (P5282; Sigma‐Aldrich) was added to the climbing piece and incubated for 1 h at about 25°C. Then, the climbing piece was blocked with fluoroshield mounting medium with DPIA (ab104139 Abcam) and visualized using a microscope.

### 2.9 Statistical analysis

Statistical analysis was performed using SPSS Statistics 23.0 software (IBM SPSS). The results are presented as means ± standard deviations. The Student's *t* test was used for comparisons between two groups. Statistical significance was set at *p* < 0.05.

## 3 RESULTS

### 3.1 MiR‐411‐3p and TGF‐β1/Smad/Smurf2 expression in BLM‐induced skin fibroblasts

**FIGURE 1:**
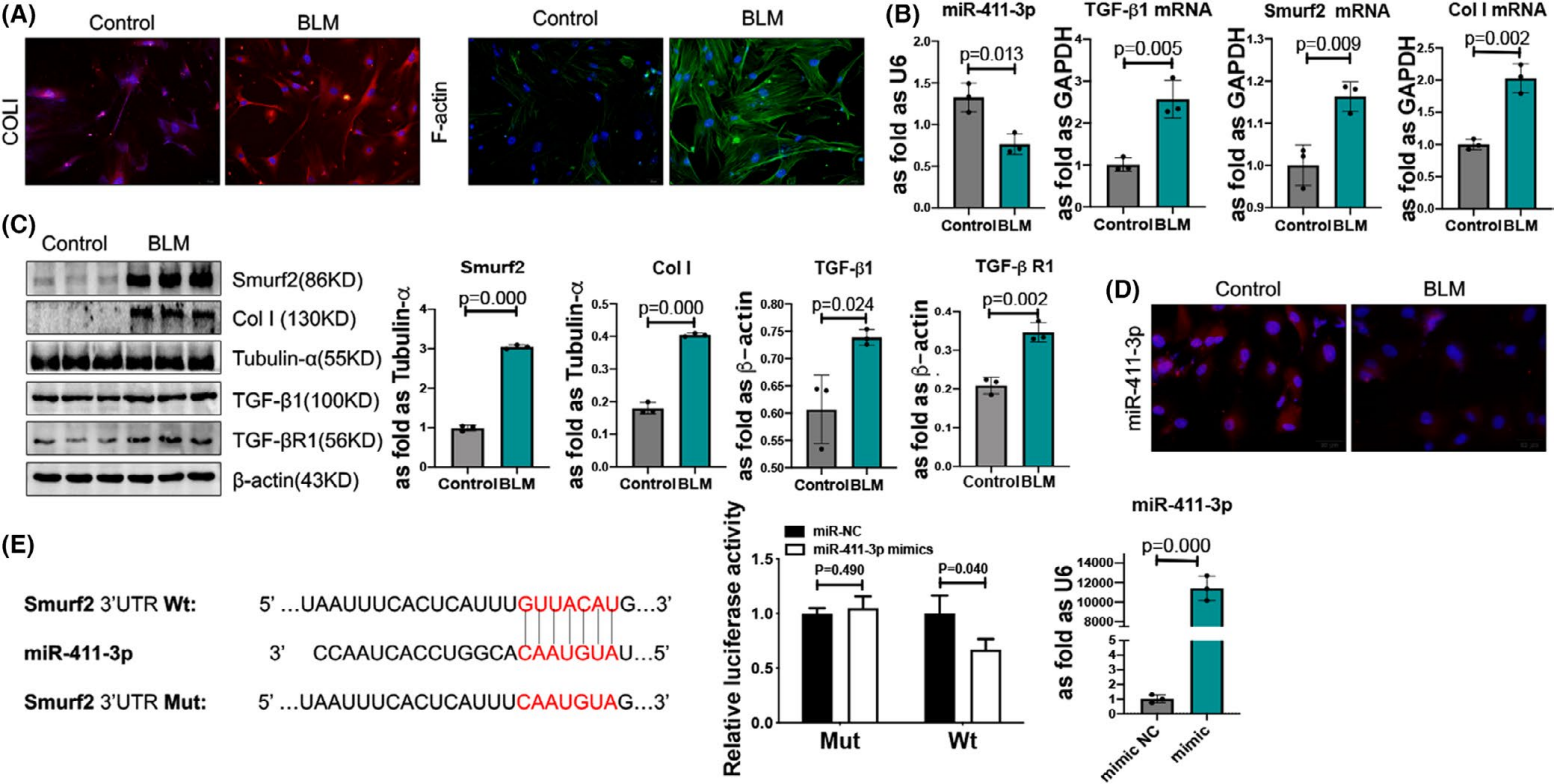
BLM accelerates the transforming growth factor (TGF)‐β1/Smad/Smad ubiquitin regulatory factor‐2 (Smurf2) signalling pathway and attenuates microRNA (miR)‐411‐3p in skin fibroblasts, meanwhile, miR‐411‐3p exerts inhibitory effects on Smad ubiquitin regulatory factor‐2 (Smurf2) in skin fibroblasts. (A) Collagen I (COLI) immunofluorescence and F‐actin phalloidin staining in skin fibroblasts (scale bar =50 μm). (B) The mRNA levels of miR‐411‐3p, TGF‐β1, Smurf2 and COLI in BLM‐induced skin fibroblasts were measured using real‐time quantitative polymerase chain reaction. (C) The protein levels of COLI, TGF‐β1, TGF‐β receptor (RI) and Smurf2 in bleomycin (BLM)‐induced skin fibroblasts were analysed using Western blotting. (D) The level and location of miR‐411‐3p were shown using in situ hybridization in BLM‐induced skin fibroblasts. (E) Human embryonic kidney 293T cells were transfected with an miR‐411‐3p mimic or negative control mimic and the Smurf2 3′‐UTR wild‐type (Wt) or mutant‐type plasmid. The luciferase activity in Smurf2‐3′‐UTR‐Wt cells was decreased following transfection with the miR‐411‐3p mimic. The miR‐411‐3p level was increased in skin fibroblasts transfected with miR‐411‐3p (data are presented as means ± standard deviations; *n *= 3 independent experiments)

Firstly, we used different concentration of BLM stimulating skin fibroblasts, the protein levels of TGF‐β1, Smurf2 and COLI were obviously increased at a concentration of 3mU/ml BLM (Figure S1A, B). As shown in Figure 1A and Figure S2(A, B), increased expression levels of COLI and F‐actin by BLM were detected in skin fibroblasts. The mRNA levels of TGF‐β1, Smurf2 and COLI were also increased in BLM‐induced skin fibroblasts (Figure 1B). Moreover, the Western blot analysis results revealed that BLM increased the expression levels of TGF‐β1, TGF‐β RI, Smurf2 and COLI in skin fibroblasts (Figure 1C). The level of miR‐411‐3p was lower than that in the control group (Figure 1B), and miR‐411‐3p was found to be decreased in the BLM‐induced skin fibroblast cytoplasm (Figure 1D and Figure S3A).

### 3.2 Attenuation of Smurf2 expression by miR‐411‐3p

Target prediction databases (http://www.targetscan.org/vert_72/) have shown that Smurf2 is a possible target gene for miR‐411‐3p, constituting a complementary pair at the gene level. The dual‐luciferase reporter assay showed that overexpression of miR‐411‐3p distinctly inhibited luciferase activity in Smurf2‐3′‐UTR‐Wt HEK293T cells. However, it had no effect on luciferase activity in Smurf2‐3′‐UTR‐Mut cells (Figure 1E). As shown in Figure 1E, the miR‐411‐3p mimic significantly upregulated miR‐411‐3p expression in skin fibroblasts.

### 3.3 Overexpression of miR‐411‐3p attenuates the TGF‐β/Smad signalling pathway and the production of COLI and F‐actin in skin fibroblasts

**FIGURE 2:**
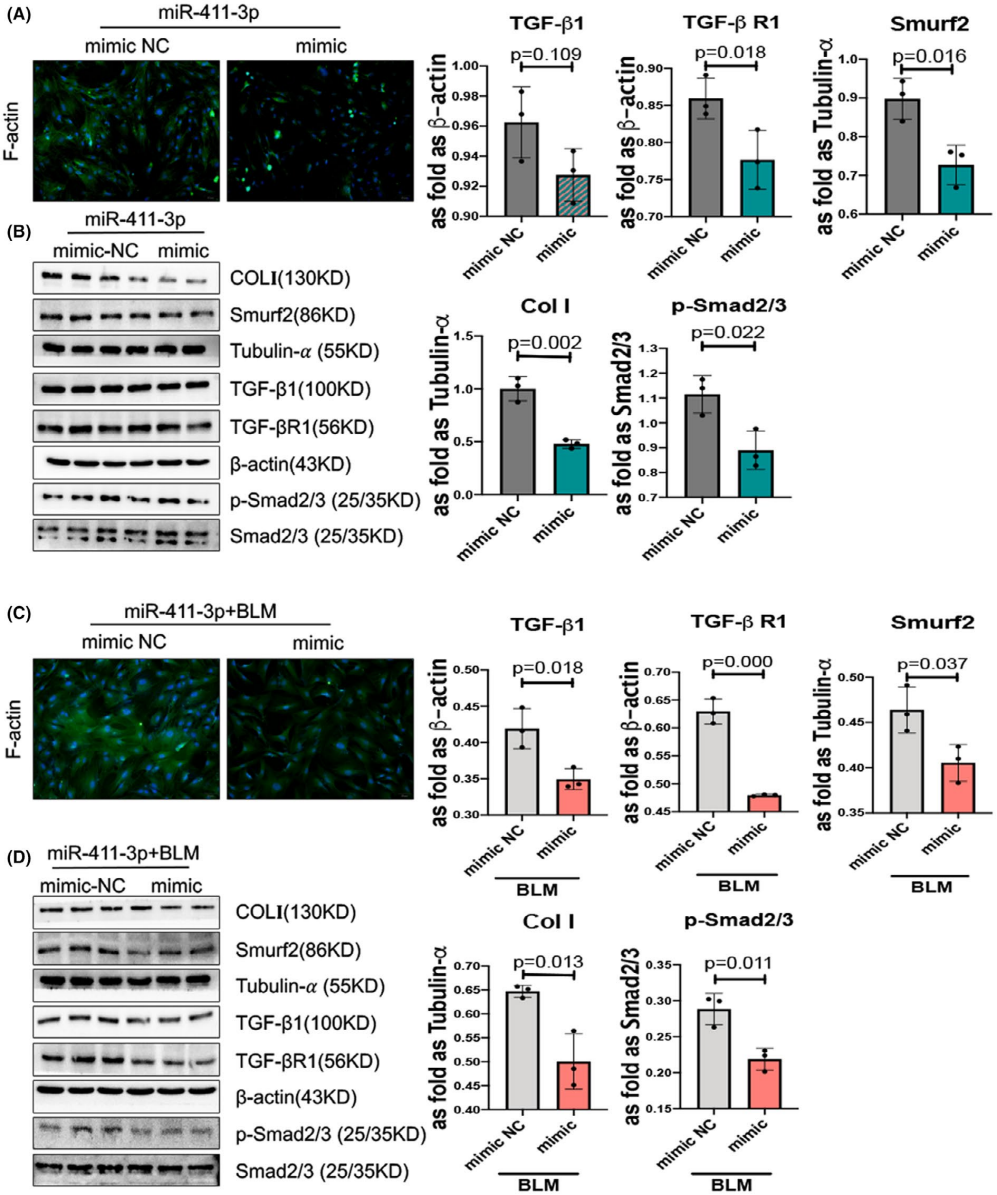
MicroRNA (miR)‐411‐3p represses transforming growth factor (TGF)‐β1/Smad/ Smad ubiquitin regulatory factor‐2 (Smurf2) signalling and F‐actin expression in skin fibroblasts. (A) Skin fibroblasts were transfected with an miR‐411‐3p mimic for 48 h. The level of F‐actin determined using phalloidin staining in miR‐411‐3p mimic‐transfected skin fibroblasts was lower than that in negative control mimic‐transfected skin fibroblasts (scale bar =50 μm). (B) The protein levels of collagen I, Smurf2, TGF‐β1, TGF‐β receptor (RI) and p‐Smad2/3 in skin fibroblasts transfected with miR‐411‐3p were analysed using Western blotting. (C) Skin fibroblasts were transfected with an miR‐411‐3p mimic for 36 h and then treated with BLM for 12 h. The phalloidin staining level of F‐actin was lower in miR‐411‐3p mimic‐transfected and BLM‐induced skin fibroblasts than in negative control mimic‐transfected skin fibroblasts (scale bar =50 μm). (D) The protein levels of collagen I, Smurf2, TGF‐β1, TGF‐β receptor (RI) and p‐Smad2/3 in miR‐411‐3p mimic‐transfected and BLM‐induced skin fibroblasts were measured using Western blotting (data are presented as means ± standard deviations; *n* = 3 independent experiments)

Figure 2A, C and Figure S4A show that the fluorescence intensity of F‐actin was reduced by the miR‐411‐3p mimic in skin fibroblasts. We found that the upregulation of miR‐411‐3p suppressed COLI, Smurf2 and positive factors in TGF‐β/Smad signalling, such as TGF‐β1, TGF‐β RI and p‐Smad2/3, in skin fibroblasts with or without BLM stimulation (Figure 2B and D).

**FIGURE 3:**
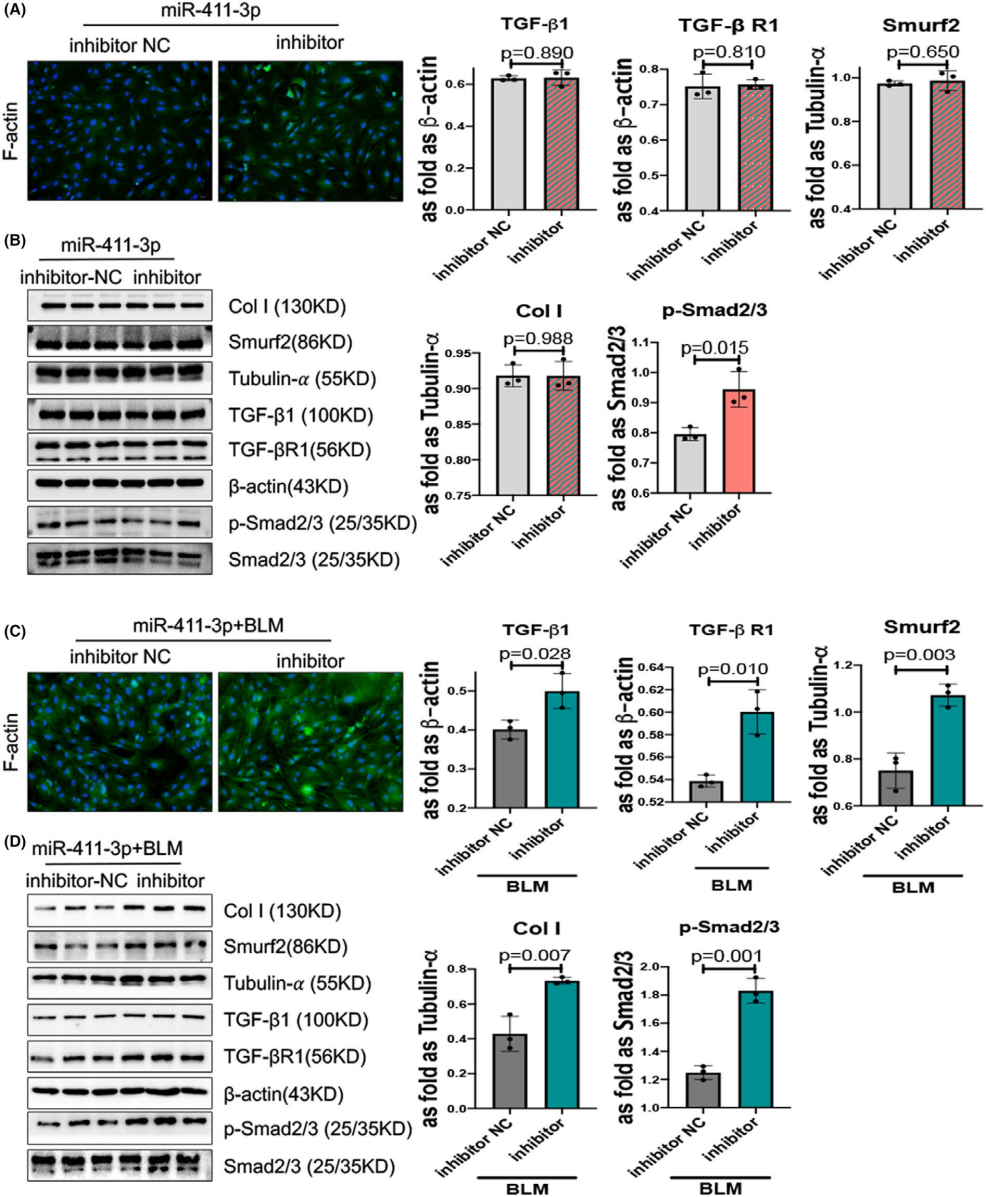
MicroRNA (miR)‐411‐3p downregulation does not increase transforming growth factor (TGF)‐β1/Smad/Smad ubiquitin regulatory factor‐2 (Smurf2) signalling in skin fibroblasts, however, increase the expression of TGF‐β1/Smad/ Smurf2 signalling and F‐actin in bleomycin (BLM)‐induced skin fibroblasts. (A) Skin fibroblasts were transfected with an miR‐411‐3p inhibitor for 48 h. The phalloidin staining level of F‐actin was increased in miR‐411‐3p inhibitor‐transfected and BLM‐induced skin fibroblasts compared with that of inhibitor NC (scale bar =50 μm). (B) The protein levels of collagen I, Smurf2, TGF‐β1, TGF‐β receptor (RI) and p‐Smad2/3 in miR‐411‐3p inhibitor‐transfected skin fibroblasts were analysed using Western blotting. (C) Skin fibroblasts were transfected with miR‐411‐3p inhibitor for 36 h and then treated with BLM for 12 h. The phalloidin staining level of F‐actin was higher in miR‐411‐3p inhibitor‐transfected and BLM‐induced skin fibroblasts than in negative control mimic‐transfected skin fibroblasts (scale bar =50 μm). (D) The protein levels of collagen I, Smurf2, TGF‐β1, TGF‐β receptor (RI) and p‐Smad2/3 in miR‐411‐3p inhibitor‐transfected and BLM‐induced skin fibroblasts were analysed using Western blotting (data are presented as means ± standard deviations; *n* = 3 independent experiments)

Furthermore, downregulation of miR‐411‐3p accelerated COLI and F‐actin hyperplasia as well as the TGF‐β/Smad signalling pathway in with or without BLM‐induced skin fibroblasts (Figure 3A, B, C, D and Figure S4B). However, the *p* value of the expression of TGF‐β/Smad signalling is not statistically significant in Figure 3B.

### 3.4 Knockdown of Smurf2 suppresses TGF‐β/Smad signalling in skin fibroblasts

**FIGURE 4:**
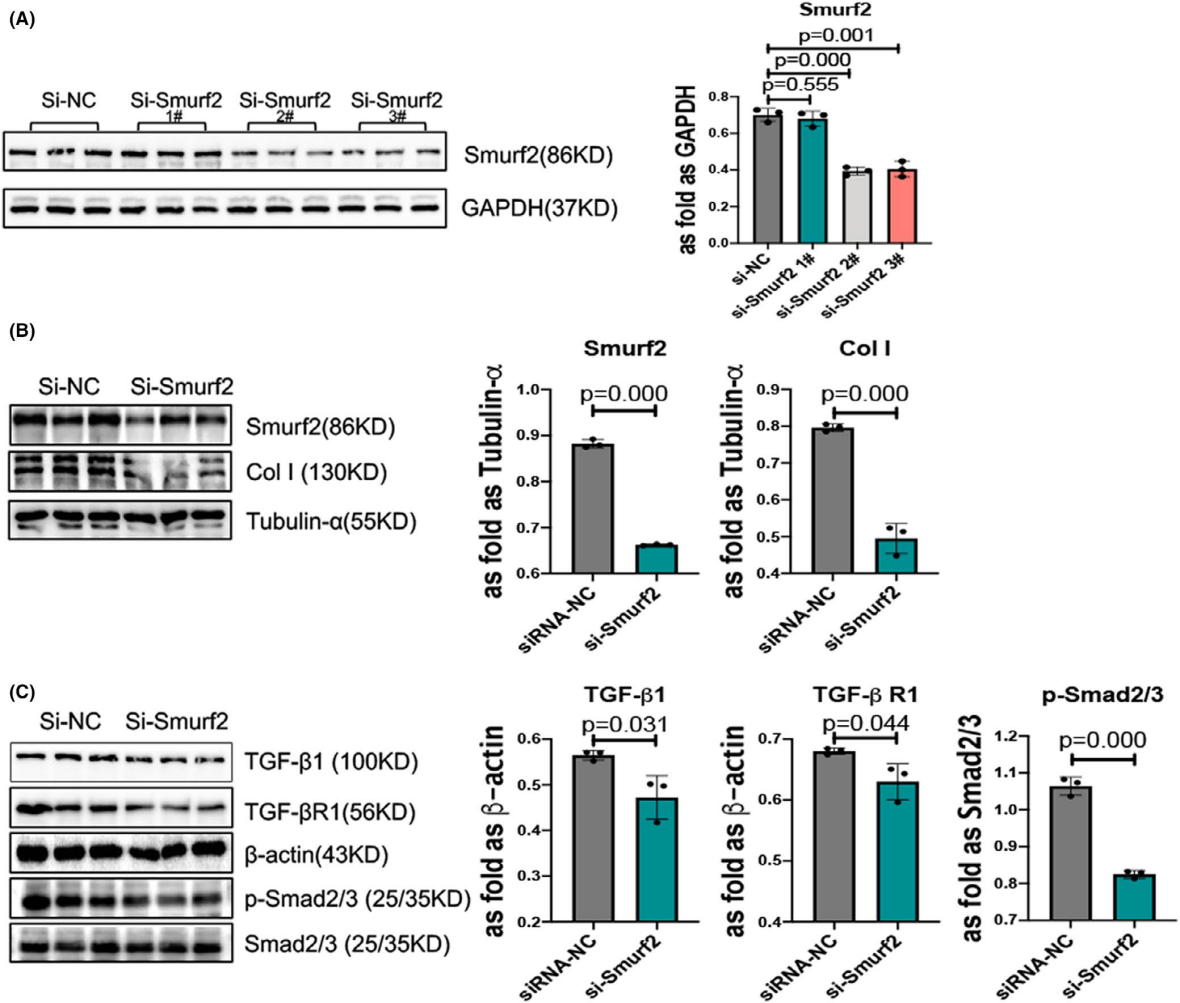
Blocking Smad ubiquitin regulatory factor‐2 (Smurf2) decreases transforming growth factor (TGF)‐β1/Smad signalling in skin fibroblasts. (A) Skin fibroblasts were transfected with small interfering RNA (siRNA)‐Smurf2 sequences. (B) The protein levels of collagen I and Smurf2 in skin fibroblasts transfected with si‐Smurf2 were analysed using Western blotting. (C) The protein levels of TGF‐β1, TGF‐β receptor (RI) and p‐Smad2/3 in skin fibroblasts transfected with si‐Smurf2 were analysed using Western blotting (data are presented as means ± standard deviations; *n* = 3 independent experiments)

To explore whether miR‐411‐3p inhibits the TGF‐β/Smad signalling pathway by downregulating Smurf2, we silenced Smurf2 using siRNA transfection (Figure 4A and B), which resulted in the downregulation of TGF‐β1, TGF‐β RI and p‐Smad2/3 (Figure 4C). In addition, the Western blot analysis results showed that the COLI level was decreased (Figure 4B).

### 3.5 MiR‐411‐3p inhibits BLM‐induced skin fibrosis by regulating TGF‐β/Smurf2/Smad

**FIGURE 5:**
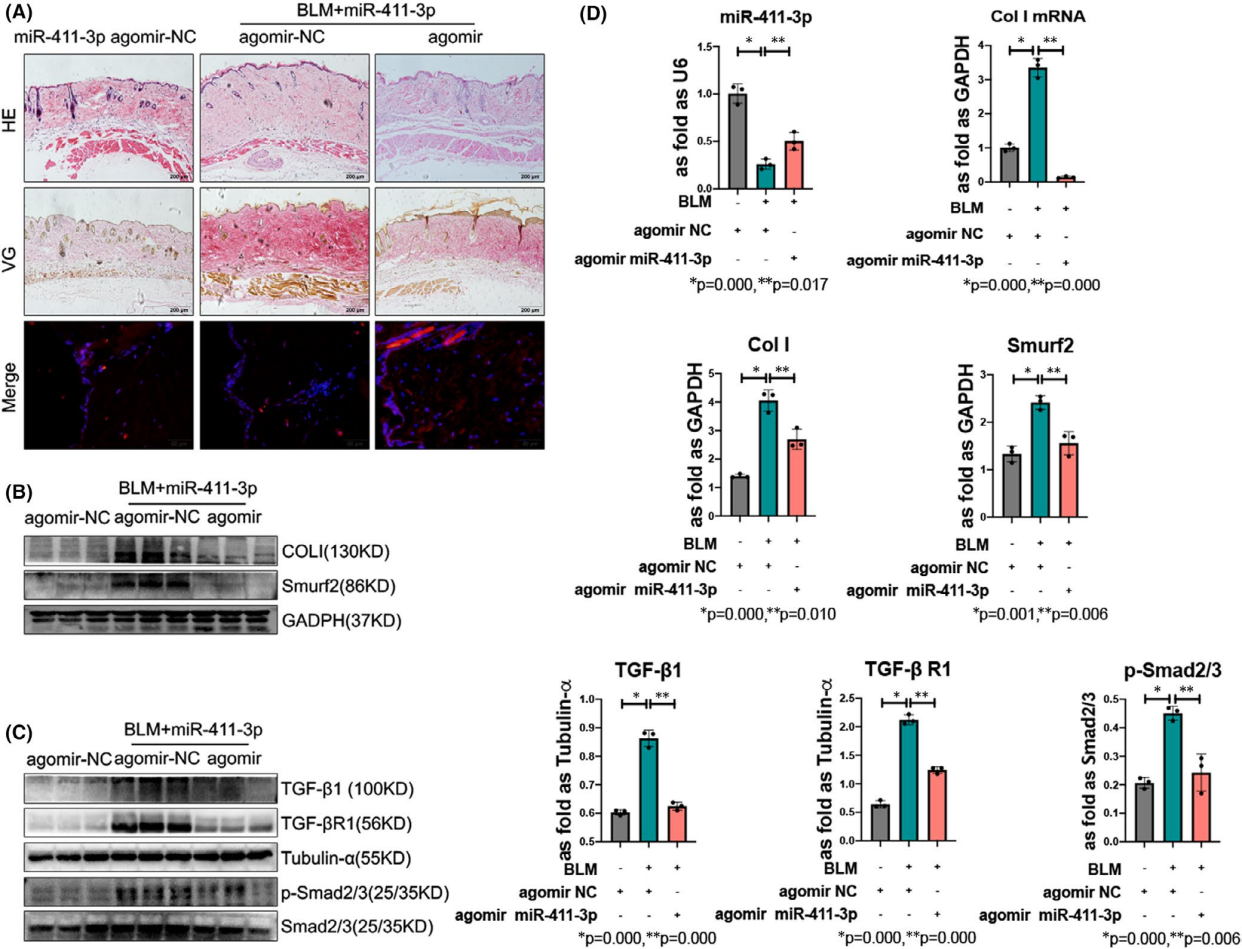
MicroRNA (miR)‐411‐3p alleviates the increased thickness and exerts inhibitory effects on transforming growth factor (TGF)‐β1/Smad signalling by targeting Smad ubiquitin regulatory factor‐2 (Smurf2) in skin fibrosis. (A) haematoxylin and eosin and Van Gieson's staining of the skin administered with miR‐411‐3p agomir (scale bar =200 μm). MiR‐411‐3p was located in the dermal layer of skin using in situ hybridization (scale bar =50 μm). (B) The protein levels of collagen I and Smurf2 in skin fibrosis were analysed using Western blotting. (C) The protein levels of TGF‐β1, TGF‐β receptor (RI) and p‐Smad2/3 in skin fibrosis were analysed using Western blotting. (D) The mRNA levels of collagen I in skin fibrosis were tested using real‐time quantitative polymerase chain reaction (data are presented as means ± standard deviations; *n* = 3 independent experiments)

Using BLM‐designed mouse skin fibrosis as a model, we found that the thickness of the dermis and subcutaneous fat was increased by histological morphology analysis, accompanied by the upregulated expression of miR‐411‐3p after delivering miR‐411‐3p agomir as an agonist (Figure 5A and Figure S3B). Western blot analysis also revealed that the levels of COLI and Smurf2 were decreased (Figure 5B). In addition, miR‐411‐3p downregulated the levels of TGF‐β1, TGF‐β RI and p‐Smad2/3 (Figure 5C). Moreover, the RT‐qPCR results demonstrated that the miR‐411‐3p level was increased and the COLI mRNA level was decreased (Figure 5D).

## 4 DISCUSSION

Skin fibrosis is one of pathological manifestations of some diseases, including keloid, hyperplastic scar and systemic sclerosis. All of these diseases possess common features, such as collagen disorder, abnormal ECM hyperplasia, fibroblast proliferation and aberrant immunity.^1^, ^2^ However, the mechanisms underlying these diseases remain unclear. In addition, there is no effective treatment for a vast number of patients.^23^ Increasing evidence has shown that miRNAs are involved in skin fibrosis.^3^, ^24^ MiR‐21 is involved in keloids and systemic sclerosis, and overexpression of miR‐21 can increase fibroblast proliferation and EMT by regulating phosphatase, tensin homolog and protein kinase B.^25^ In addition, TGF‐β1 promotes the expression of miR‐21, resulting in the inhibition of cell apoptosis and the acceleration of proliferation.^25^ Huang et al. found that in a mouse model of rheumatoid arthritis, upregulated miR‐411 inhibited the proliferation and promoted the apoptosis of synoviocytes by inhibiting nuclear factor‐κB.^26^ In a previous study, we applied high‐throughput sequencing to 70 miRNA variations, one of which was significantly downregulated in rat silicosis: miR‐411‐3p.^27^

In this study, we found that miR‐411‐3p was decreased while TGF‐β1 was increased in BLM‐induced skin fibrosis and fibroblasts. Furthermore, the expression levels of COLI and F‐actin were elevated, which may be due to TGF‐β1 induced by BLM. As previously established, TGF‐β1 is one of the widely recognized factors that can promote fibrosis.^13^ In skin fibrosis, the proliferation of dermal fibroblasts and collagen accumulation is regulated by the upregulated TGF‐β1/Smad signalling pathway.^28^ Smurf2 plays an important role in TGF‐β signalling as a key regulatory factor in the ubiquitination pathway. In the present study, Smurf2 was increased in mice skin fibrosis and fibroblasts induced by upregulated TGF‐β1. Furthermore, Smurf2, as a regulator, exerts a different function on the TGF‐β1/Smad signalling pathway.^15^, ^29^ Several studies have demonstrated that Smurf2 is regulated by certain miRNAs. For example, in intestinal epithelial cells, miR‐322 and miR‐503 affect homeostasis and cell apoptosis by repressing Smurf2 translation, which induces the degradation of phosphorylated Smad2.^30^ Overexpression of miR‐485 has been observed to decrease the expression of α‐SMA and inhibits ASMC proliferation by repressing the levels of TGF‐β1 and Smad3 via Smurf2 downregulation.^20^ In pancreatic cancer cells, miR‐15b downregulate Smurf2 expression, attenuating the inhibition of TGF‐β to promote EMT.^31^ In addition, upregulation of miR‐411‐3p inhibits the levels of Smurf2 mRNA and translation, leading to COLI and α‐SMA expression in silicosis.^11^ However, in skin fibrosis, the mechanisms of Smurf2 and miR‐411‐3p, which regulate the TGF‐β1/Smad signalling pathway, are not clear.

Here, we performed a dual‐luciferase reporter assay and found that the 3′‐UTR of Smurf2 binds to miR‐411‐3p. We administered miR‐411‐3p agomir or mimic both in vitro and in vivo and found that overexpression of miR‐411‐3p inhibited COLI hyperplasia by negatively regulating Smurf2. Moreover, our results demonstrated that the expression of TGF‐β RI and p‐Smad2/3 was downregulated by blocking Smurf2. In summary, downregulation of Smurf2 can attenuate the ubiquitination/proteasomal degradation of TGF‐β RI in skin fibroblasts and skin fibrosis induced by BLM. In skin fibrosis, overexpression of miR‐411‐3p exerts a suppressive effect on Smurf2, which may inhibit the TGF‐β/Smad signalling pathway by decreasing the expression of TGF‐β RI.

## CONFLICT OF INTEREST

The authors declare they have no competing interests.

## AUTHOR CONTRIBUTIONS

**Ziyan Zhang:** Conceptualization (lead); Data curation (lead); Formal analysis (lead); Methodology (lead); Resources (lead); Software (lead); Writing‐original draft (lead); Writing‐review & editing (lead). **Xuemin Gao:** Data curation (supporting); Resources (supporting). **Yang He:** Data curation (supporting). **Yumeng Kang:** Data curation (supporting). **Fuyu Jin:** Resources (supporting). **Yaqian Li:** Resources (supporting). **Tian Li:** Resources (supporting). **Zhongqiu Wei:** Data curation (supporting). **Shifeng Li:** Data curation (supporting). **Wenchen Cai:** Data curation (supporting); Formal analysis (supporting). **Na Mao:** Data curation (supporting); Formal analysis (supporting). **Shan Wang:** Data curation (supporting). **Heliang Liu:** Supervision (supporting). **Fang Yang:** Funding acquisition (lead); Project administration (lead); Validation (lead). **Hong Xu:** Funding acquisition (lead); Project administration (lead); Supervision (lead); Validation (lead). **Jie Yang:** Supervision (lead); Validation (lead).

## Supporting information

Supplementary Material
