# Supplementary material for: MicroRNA‐411‐3p inhibits bleomycin‐induced skin fibrosis by regulating transforming growth factor‐β/Smad ubiquitin regulatory factor‐2 signalling
Source: J Cell Mol Med. 2021 Nov 15;25(24):11290–9. doi: 10.1111/jcmm.17055 (PMC8650044; doi:10.1111/jcmm.17055)
Supplement: Supplementary file 1 — Supplementary Material [file JCMM-25-11290-s001.docx]

**Supplementary figure legends**

**
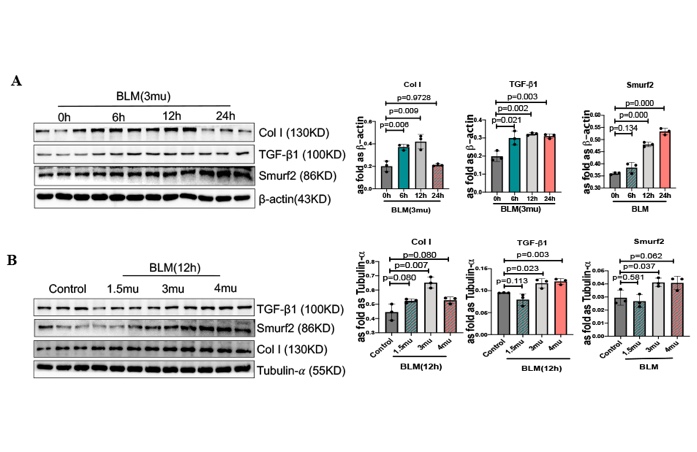
**

**Supplementary Figure 1 Bleomycin (BLM) induces skin fibroblasts in time- and concentration-dependent manners.** (A) The protein levels of transforming growth factor (TGF)-β1, Smad ubiquitin regulatory factor-2 (Smurf2), and collagen I (COLI) in skin fibroblasts induced by BLM over time were analyzed using western blotting. (B) The protein levels of TGF-β1, Smurf2, and COLI in skin fibroblasts induced by BLM with different concentrations were measured using western blotting (data are presented as means ± standard deviations; n = 3 independent experiments).


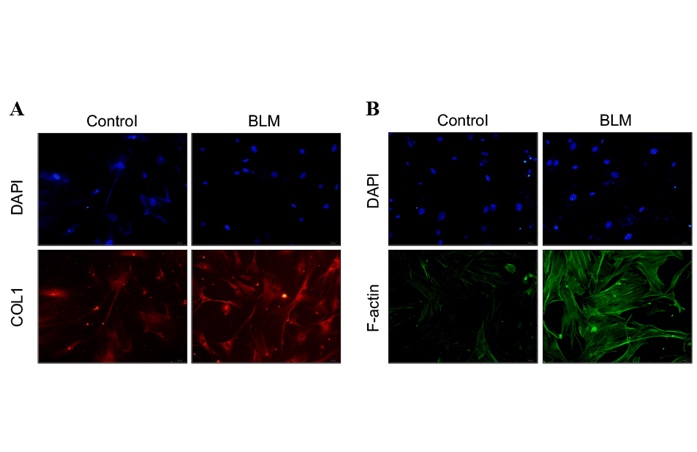


**Supplementary Figure 2 Bleomycin (BLM) promotes collagen I (COLI) and F-actin expression in skin fibroblasts.** (A) COLI immunofluorescence in skin fibroblasts induced by BLM (bar = 50 μm). (B) F-actin phalloidin staining in skin fibroblasts induced by BLM (bar = 50 μm; data are presented as means ± standard deviations; n = 3 independent experiments).

**
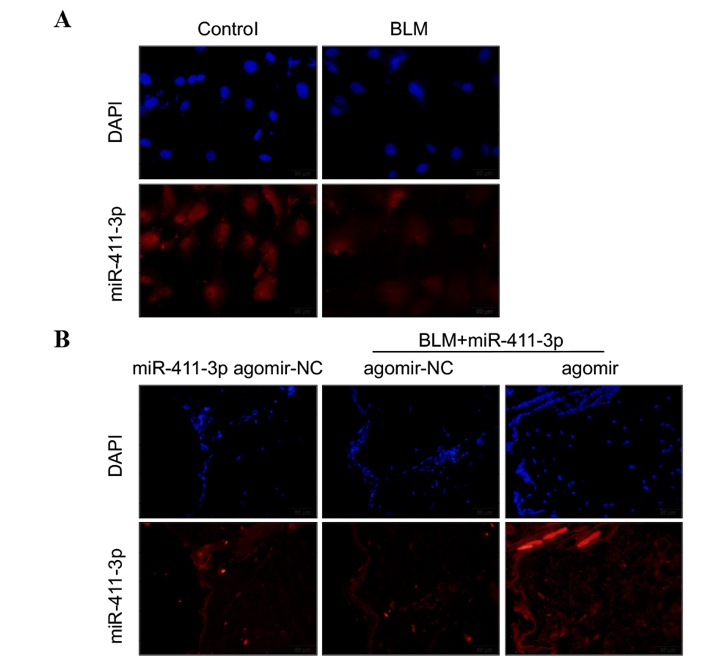
**

**Supplementary Figure 3 Location of microRNA (miR)-411-3p in the skin and skin fibroblasts.** (A) The location of miR-411-3p was shown in the cytoplasm of fibroblasts using *in situ* hybridization (scale bar = 50 μm); (B) MiR-411-3p was located in the dermal layer of the skin as measured by *in situ* hybridization (scale bar = 50 μm; data are presented as means ± standard deviations; n = 3 independent experiments).

**
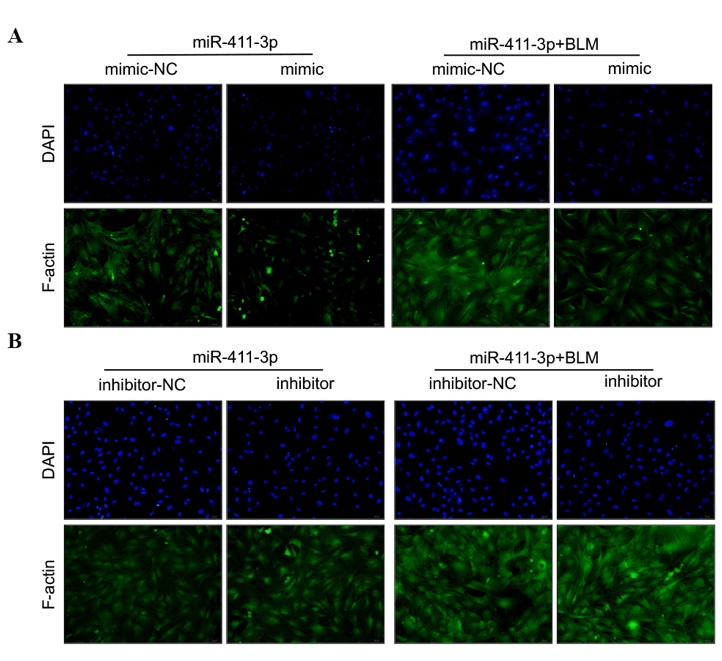
**

**Supplementary Figure 4 MicroRNA (miR)-411-3p regulates F-actin expression in skin fibroblasts.** (A) F-actin phalloidin staining in skin fibroblasts via transfection of an miR-411-3p mimic with or without bleomycin (BLM) induction (scale bar = 50 μm); (B) F-actin phalloidin staining in skin fibroblasts transfected with an miR-411-3p inhibitor with or without BLM induction (scale bar = 50 μm; data are presented as means ± standard deviations; n = 3 independent experiments).

**Supplementary table**

**Table S1 The sequences of miRNAs and siRNAs**

| Mimic/inhibitor/siRNA | Sense (5’-3’) | Antisense (5’-3’) |
| --- | --- | --- |
| mmu-miR-411-3p agomir | UAUGUAACACGGUCCACUAACC | GGUUAGUGGACCGUGUUACAUA |
| agomir Negative Control | UUUGUACUACACAAAAGUACUG | CAGUACUUUUGUGUAGUACAAA |
| mimic Negative Control | UUUGUACUACACAAAAGUACUG | CAGUACUUUUGUGUAGUACAAA |
| mmu-miR-411-3p mimic | UAUGUAACACGGUCCACUAA | UUAGUGGACCGUGUUACAUA |
| inhibitor Negative Control | CAGUACUUUUGUGUAGUACAAA | - |
| mmu-miR-411-3p inhibitor | UUAG UGGACCGUGUUACAUA | - |
| siRNA-Negative Control | UUCUCCGAACGUGUCACGUdTdT | ACGUGACACGUUCGGAGAAdTdT |
| Si-Smurf2_001 | GGUGCUGGAUUUCUUGGUUdTdT | AACCAAGAAAUCCAGCACCdTdT |
| Si-Smurf2_002 | CCAGAUGGUUGGGAAGAAAdTdT | UUUCUUCCCAACCAUCUGGdTdT |
| Si-Smurf2_003 | GCAGGGCUUCAAAGCACUAdTdT | UAGUGCUUUGAAGCCCUGCdTdT |
